# Assessing Continuous Epidural Infusion and Programmed Intermittent Epidural Bolus for Their Effectiveness in Providing Labor Analgesia: A Mono-Centric Retrospective Comparative Study

**DOI:** 10.3390/medicina59091579

**Published:** 2023-08-30

**Authors:** Shao-Lun Tsao, Wen-Tyng Li, Li-Yun Chang, Pin-Hung Yeh, Liang-Tsai Yeh, Ling-Jun Liu, Chao-Bin Yeh

**Affiliations:** 1 Department of Anesthesiology, Changhua Christian Hospital, Changhua 500, Taiwan; 2 Department of Biomedical Engineering, Chung Yuan Christian University, Taoyuan City 320, Taiwan; 3 Institute of Medicine, Chung Shan Medical University, Taichung 402, Taiwan; 4 Department of Post-Baccalaureate Medicine, College of Medicine, National Chung Hsing University, Taichung 402, Taiwan; 5 Department of Statistics, Tung Hai University, Taichung 407, Taiwan; 6 School of Medicine, Chung Shan Medical University, Taichung 402, Taiwan; 7 Department of Emergency Medicine, Chung Shan Medical University Hospital, Taichung 402, Taiwan

**Keywords:** analgesia, labor pain, workload, anesthesia, epidural, patient-controlled

## Abstract

*Background and Objectives*: Local anesthetics administered via epidural catheters have evolved from intermittent top-ups to simultaneous administration of continuous epidural infusion (CEI) and patient-controlled epidural analgesia (PCEA) using the same device. The latest programmed intermittent epidural bolus (PIEB) model is believed to create a wider and more even distribution of analgesia inside the epidural space. The switch from CEI + PCEA to PIEB + PCEA in our department began in 2018; however, we received conflicting feedback regarding workload from the quality assurance team. This study aimed to investigate the benefits and drawbacks of this conversion, including the differences in acute pain service (APS) staff workload, maternal satisfaction, side effects, and complications before and after the changeover. *Materials and Methods*: Items from the APS records included total delivery time, average local anesthetic dosage, and the formerly mentioned items. The incidence of side effects, the association between the duration of delivery and total dosage, and hourly medication usage in the time subgroups of the CEI and PIEB groups were compared. The staff workload incurred from rescue bolus injection, catheter adjustment, and dosage adjustment was also analyzed. *Results*: The final analysis included 214 and 272 cases of CEI + PCEA and PIEB + PCEA for labor analgesia, respectively. The total amount of medication and average hourly dosage were significantly lower in the PIEB + PCEA group. The incidences of dosage change, manual bolus, extra visits per patient, and lidocaine use for rescue bolus were greater in the PIEB + PCEA group, indicating an increased staff workload. However, the two groups did not differ in CS rates, labor time, maternal satisfaction, and side effects. *Conclusions*: This study revealed that while PIEB + PCEA maintained the advantage of decreasing total drug doses, it inadvertently increased the staff burden. Increased workload might be a consideration in clinical settings when choosing between different methods of PCEA.

## 1. Introduction

Labor and delivery can cause moderate to severe pain [1]. Numerous treatments for relieving labor pain are currently available, including nonpharmacological and pharmacological methods. Nonpharmacological approaches are present, such as relaxation, breathing methods, hydrotherapy, and aromatherapy [2,3,4]. Some pharmacological approaches include intramuscular meperidine, intravenous patient-controlled analgesia with ultra-short-acting opioids, spinal morphine, and neuraxial block (e.g., epidural, combined spinal epidural, dural puncture epidural) with local anesthetics [5,6,7]. Although various innovative pain management treatments for labor analgesia are constantly being presented, epidural analgesia with long-acting local anesthetics and low-dose opioids is still regarded as the gold standard of care [1,5,8].

Methods of delivering local anesthetics via epidural catheters have evolved over time. It has progressed from intermittent top-up to continuous infusion using a device that simultaneously provides continuous epidural infusion (CEI) and patient-controlled epidural analgesia (PCEA) [8,9]. Since its introduction in 1988 by Gambling et al., the use of CEI with the PCEA model has become the mainstream approach to achieving painless labor. This approach offers several advantages, including minimizing the required local anesthetic dosage and reducing the clinical burden, whilst improving maternal satisfaction [10]. Compared with CEI, the novel programmed intermittent epidural bolus (PIEB) model provides local anesthetics at planned intervals with a higher injection pressure, which causes a wider and more optimal distribution within the epidural space [11]. Several studies have revealed that the PIEB + PCEA regimen is beneficial in lowering labor pain, reducing local anesthetics, reducing motor block, decreasing instrumental delivery, and boosting maternal satisfaction when compared to the CEI + PCEA regimen [9,11,12].

In recent years, an increasing number of commercial devices equipped with PIEB and PCEA regimens have become available. After analyzing relevant information in the literature, the Acute Pain Service (APS) committee in our department agreed to switch from CEI + PCEA to PIEB + PCEA in October 2018. The preparatory work for the conversion included serial anesthetic staff instruction (e.g., device settings, troubleshooting, safety issues) and frequent communication with obstetrics and delivery room nurses for approximately two months. However, after the conversion, some contentious inputs about workload continued to emerge from the quality assurance committee and APS workers, even one year later. Therefore, the goal of this study was to investigate the differences before and after the changeover of the epidural regimen, regarding APS staff workload, maternal satisfaction, side effects, caesarian section rate, delivery time, and average local anesthetics dosage.

## 2. Materials and Methods

### 2.1. Data Sources

This study was approved by the Human Research Ethics Committee of the Institutional Review Board of Changhua Christian Hospital (reference number: 211,221). The requirement for informed consent was waived owing to the retrospective nature of this cohort study. The study design followed the STROBE standards for observational studies [13]. To eliminate additional vigilance from the new introduction, the inclusion eligibility criteria were full-term primiparas and multiparas with ASA (American Society of Anesthesiologists) physical status I-II, aged between 18 and 45, receiving epidural labor analgesia before conversion (July 2018 to September 2018) and one year after conversion (November 2019 to January 2020). The exclusion criteria were patients with ASA III-IV, a pregnancy term other than 36–40 weeks, hypersensitivity, and contraindications to neuraxial analgesia, inadequate analgesia, reinsertion of the epidural catheter, deliberate dural puncture, combined spinal epidural (CSE), and intentional dural puncture epidural method.

### 2.2. Study Group and Outcome Measurement

The indications at the start of labor epidural analgesia were the pregnant women who were admitted to the labor ward by obstetricians, with regular uterine contraction or labor signs. Labor epidural analgesia was administered only by attending physicians or senior residents using an 18-gauge Tuohy needle and a 20-gauge catheter (Perifix^®^ B. Braun Melsungen AG, Melsungen, Germany). Levobupivacaine 0.125% with fentanyl 1.25 μg/mL was used for maintenance in both CEI + PCEA and PIEB + PCEA. In the CEI + PCEA group, the constant flow rate was 6–10 mL/h, and PCEA was 6–10 mL with a 30 min lockout interval. In the PIEB + PCEA group, the protocol for PIEB was 3–6 mL every 30 min, and PCEA was 3–6 mL at 15 min post-PIEB or post-PCEA. The pump flow rate of the PIEB bolus was 250 mL/h. To enhance the spread of the drugs to the sacral nerve roots after late first-stage labor, patients were positioned with a 30–40° elevation of the head off the bed when the cervical os ≥ 6 cm. If breakthrough pain was not effectively controlled, lidocaine 1% (5–10 mL) was administered as a manual rescue bolus. The period evaluated was from the insertion of an epidural catheter in the labor room to the removal of the epidural catheter in the postpartum recovery room.

If side effects (e.g., dizziness, nausea, vomiting, itching, drowsiness) happened, attending physicians or senior residents would assess its severity and correlation with epidural medications, and gave treatment accordingly. If caesarean section following epidural labor analgesia was needed, our standard dose of EA was 2% lidocaine with epinephrine (1:200,000) 15 to 25 mL and fentanyl 0.1 mg.

Information was gathered from the digital APS management system, quality assurance database, APS care diary, and hospital chart records. Demographic characteristics including age, height, weight, body mass index (BMI), primipara or multipara, cervical os, gestational age, site of needle insertion, depth from the skin to the epidural space, and depth of catheter insertion were collected. The key evaluations of extracted data were comparisons of the incurred clinician workload, and incidence and severity of side effects between the groups (CEI or PIEB). The incurred workload included the frequency of catheter adjustment, dosage adjustment, manual top-ups, extra visits per patient, and medicine usage for manual rescue boluses. The severity of side effects was graded by clinical personnel on a 4-point Likert scale, where 0 indicated no side effects, and 1, 2, and 3 indicated mild, moderate, and severe side effects, respectively. The incidence of complications was also analyzed according to different weight statuses. Participants with BMI under 18.5 were defined as “underweight”, 18.6–24.9 as “standard weight”, 25–29.9 as “overweight”, and >30 as “obesity”. Routine check-ups were performed every 2 h to guarantee the quality of care and resolve mechanical faults; however, sometimes staff made extra visits to respond to parturient complaints. Therefore, extra visits were defined as times a parturient required staff attention, apart from a standard checkup. Other evaluations of extracted data included the rate of conversion of scheduled normal spontaneous delivery (NSD) to caesarean section (CS) due to prolonged labor or any other reason, total delivery time from the first to the third stage of labor, total and hourly medication usage from CEI or PIEB devices, and maternal satisfaction score. Maternal satisfaction scores ranged from 1 to 5 points, with 1 point representing “severe dissatisfaction” and 5 representing “very satisfied”.

### 2.3. Plans of Statistical Analysis

The demographic factors were compared between the CEI + PCEA and PIEB + PCEA groups using independent *t*-tests. The staff workload was calculated using the average frequency of the following three tasks: bolus injection, catheter adjustment, and dose adjustment. The severity and incidence of side effects were compared between the two groups. The odds ratios were calculated. Incidence of complications among participants with different weight statuses was compared using ANOVA. The rates of altering scheduled NSD to CS, maternal satisfaction, and total delivery time were compared using independent *t*-tests. In both groups, total delivery time was compared between participants with early analgesia and the rest. Early analgesia was defined as those who received the epidurals when OS was less than 3 cm. A 2 (PIEB vs. CEI) × 2 (early OS vs. normal OS) ANOVA was administered to compare the total delivery time. Relationships between the duration of delivery and the total dosage were calculated using Pearson’s correlation. CEI and PIEB were further subdivided into five subgroups based on the length of delivery time: <4 h (fast), 4–8 h (fast-to-average), 8–16 h (average), 16–24 h (average-to-slow), and >24 h (slow). A two-way analysis of variance (ANOVA) was used to compare hourly medication usage between the CEI and PIEB subgroups.

## 3. Results

### 3.1. Characteristics of the Participants

**Figure 1:**
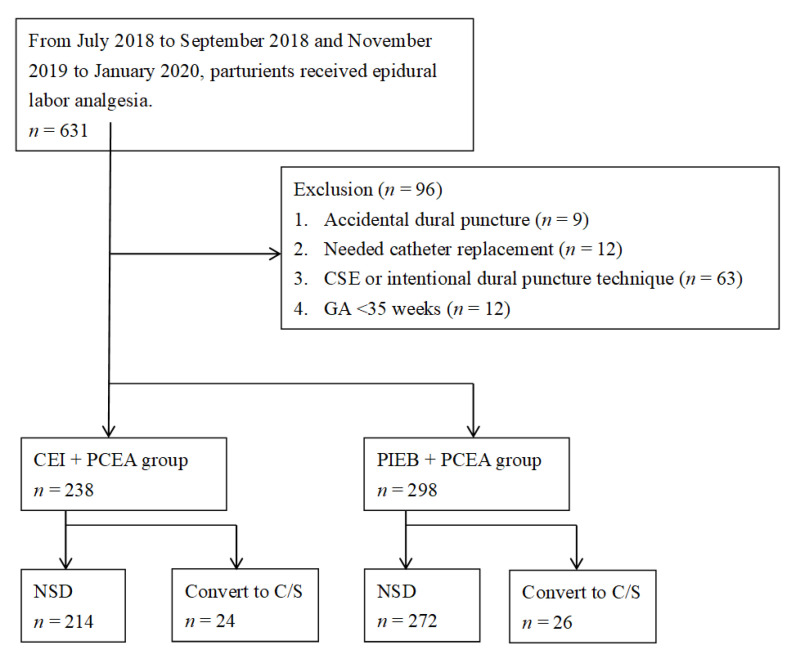
Study workflow. Abbreviations: continuous epidural infusion (CEI); patient-controlled epidural analgesia (PCEA); programmed intermittent epidural bolus (PIEB); normal spontaneous delivery (NSD); caesarean section (CS).

This study enrolled 631 parturients, 283 in the CEI + PCEA group and 384 in the PIEB + PCEA group. A total of 96 parturients were excluded from the study (nine experienced an accidental dural puncture, 12 needed catheter replacement, 12 had gestational age <35 weeks, and 63 used CSE or intentional dural puncture technique). Caesarean delivery was performed in 24 of the remaining 238 parturients (10.1%) in the CEI + PCEA group due to extended labor or decreased fetal heart rhythm, whereas 26 of the remaining 298 parturients (8.4%) in the PIEB + PCEA underwent caesarean delivery (*p* > 0.05). The final study comprised 214 parturients who underwent CEI + PCEA and 272 who underwent PIEB + PCEA to maintain painless labor. The workflow of this study is shown in Figure 1.

**Figure 2:**
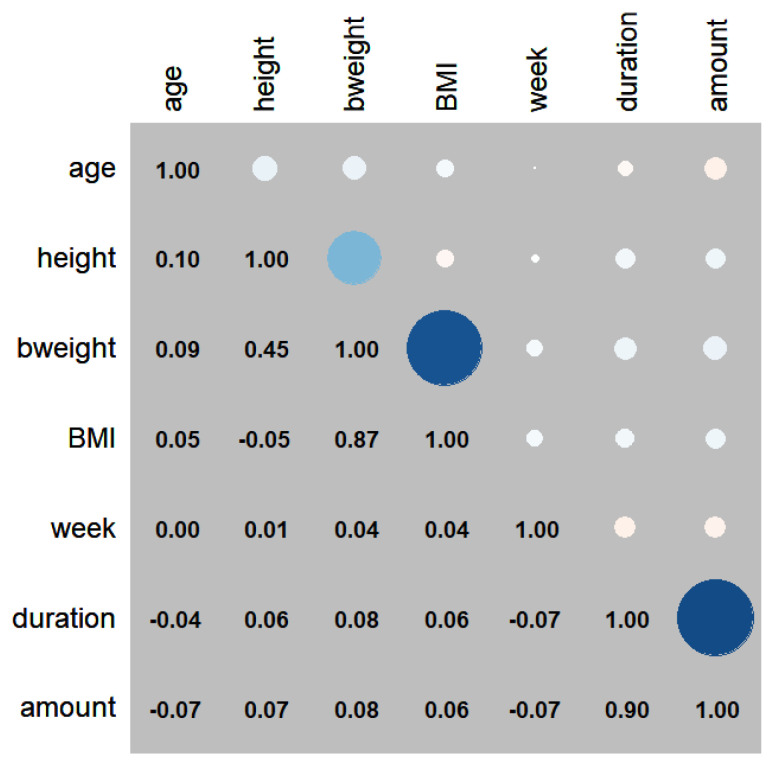
Correlations between demographic characteristics. The larger and deeper color of the circles indicated stronger correlations.

The demographic characteristics of age, height, weight, BMI, gestational age, primipara or multipara, cervical os, epidural puncture level, depth of skin to epidural or catheter insertion, and catheter fixation mark were compared between CEI and PIEB; there was no statistical difference between the two groups except for the depth of catheter insertion (6.2 cm vs. 6.0 cm, *p* < 0.05) (Table 1). Figure 2 depicts the correlations between demographic characteristics, total delivery duration, and the total amount of medication used.

### 3.2. Comparative Analysis of Epidural Analgesia Techniques

**Figure 3:**
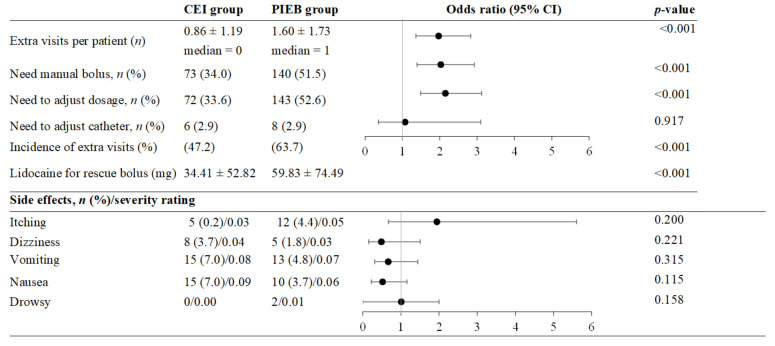
Frequency of extra workload, dose of rescue bolus, severity of the side effects, and frequency of side effects.

Given the sample size, an examination of the difference in additional work incidence rates between CEI + PCEA and PIEB + PCEA showed a power higher than 0.96. In terms of primary outcomes, the CEI + PCEA and PIEB + PCEA groups experienced comparable rates of epidural catheter adjustment (2.9% and 2.9%, respectively); however, the PIEB + PCEA group presented a greater frequency of dosage adjustment (33.6% in CEI + PCEA vs. 52.6% in PIEB + PCEA, *p* < 0.001), manual bolus administration (34% vs. 51.5%, *p* < 0.001), and additional visits per patient (0.86 vs. 1.60, *p* < 0.001). Lidocaine utilized for the rescue bolus was also higher in the PIEB + PCEA group (34.41 vs. 59.83 mg, *p* < 0.001). The incidence rates of adverse effects were not significantly different across the groups (*p* > 0.15) (Figure 3). There was no group effect in the comparison of complication incidences among participants with different weight statuses (all ps > 0.301).

### 3.3. Analysis for Perinatal Pain Management between PIEB and CEI

**Figure 4:**
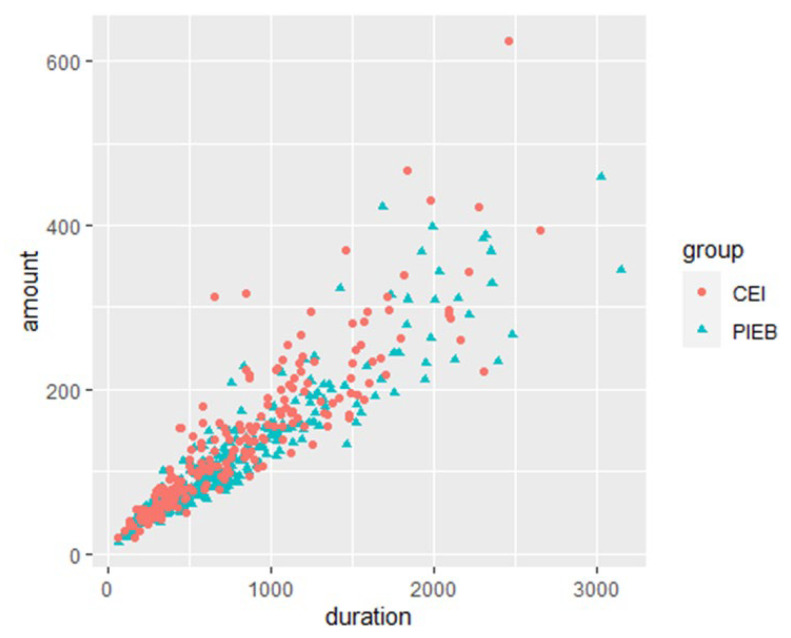
Medication scatter plots in CEI (pink color) and PIEB (green color). The graph reveals an outlier, indicating that one of the CEI patients received an excessive amount of medication.

**Figure 5:**
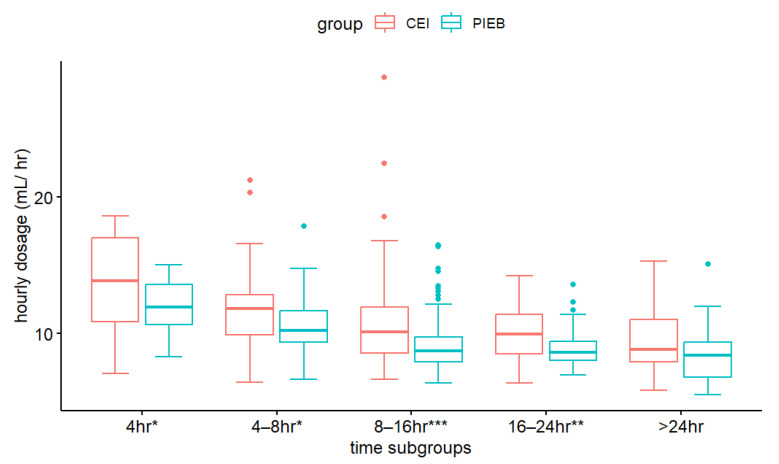
The hourly average medication consumption in CEI (pink) and PIEB (green). * *p* < 0.05, ** *p* < 0.01, *** *p* < 0.001.

The percentage of switching from NSD to CS, the overall duration of delivery, and maternal satisfaction were comparable across CEI and PIEB (*p* ≥ 0.32). There was no interaction between analgesia groups and OS groups on the overall duration of delivery. OS showed a significant main effect (F = 79.53, *p* < 0.001). Overall duration of delivery was significantly longer in early analgesia, regardless of the method of PIEB or CEI. The total amount of medication administered was considerably lower in the PIEB + PCEA group (*p* = 0.01). The average hourly dose was significantly lower in the PIEB + PCEA group (*p* < 0.001) (Table 2). Figure 4 depicts scatter plots of the drug quantities. A two-way ANOVA was used to compare the average hourly dosage in the PIEB and CEI time segments. The results revealed a substantial group impact (F = 41.56, *p* < 0.001) and a significant time effect (F = 22.61, *p* < 0.001) on average hourly dosage. The interaction between the group and time was not significant (*p* = 0.53). Further investigation revealed that in patients with total delivery times of <4, 4–8, 8–16, and 16–24 h, the average hourly dosage differed between the PIEB and CIE groups (Table 3). The PIEB group consistently consumed lower average hourly doses during these time segments (Figure 5).

## 4. Discussion

Our study found that PIEB combined with PCEA increased the workload of the anesthetic staff regarding extra visits, device dosage adjustment, and manual bolus administration when compared to CEI combined with PCEA. PIEB combined with PCEA required less local anesthetics and opioids during the three labor phases. The two groups had comparable CS rates, labor time, maternal satisfaction, and side effects.

We also revealed that the average dosage per hour was inversely associated with labor time. When the labor time was <4 h, the PIEB group required up to 12 mL/h; however, when the labor time was >24 h, the PIEB group only required 8.53 mL/h. Although the usual labor time is 8–16 h, competent obstetric staff can estimate the speed of labor progression from characteristics such as higher parity, lower fetal weight, and spontaneous rupture of the membranes [14,15]; therefore, it is reasonable to consult obstetric nurses or obstetricians about the expected progression and alter the starting dosage accordingly. The current study’s time subgroups analysis suggested that if the estimated delivery time is <8 h, a higher starting dosage (around 10.66 mL/h) may help achieve optimal pain control during labor; if the estimated delivery time is >16 h, the clinician should start with a lower dosage (~8.87 mL/h). Providing appropriate analgesics from the start may assist in reducing the nursing effort required for additional bedside visits. However, it should also be noted that some patients have a high pain threshold, and the fixed concentration may not be able to give sufficient analgesia during the accelerated phase. Appropriate increases in local anesthetic concentration may need to be considered in some patients.

Previous studies have shown that PIEB via a multiport epidural catheter can cause more sensory block than CEI. In the PIEB group, fewer medications were required, resulting in fewer side effects and motor blocks [16,17]. In our study, we revealed that the PIEB group used less medicine overall, but there were no significant differences in side effects between the two groups. We hypothesized that this was caused by poor PIEB dosage or lockout interval, requiring parturients to use more rescue lidocaine for breakthrough pain, and reducing the benefits of PIEB on the occurrence of adverse effects.

According to Kanczuk et al., the optimal time interval between PIEBs of 10 mL of bupivacaine 0.0625% with fentanyl 2 μg/mL, which can offer adequate analgesia for 90% of females throughout their first stage of labor, is 42.6 min [18]. Furthermore, the motor blockage was less noticeable in the PIEB groups at 40, 50, and 60 min intervals than in the PIEB group at 30 min intervals, although the incidence of hypotension was equivalent between groups with no therapy required [18]. Our regimen was 3–6 mL of levobupivacaine 0.125% with fentanyl 1.25 μg/mL and a time interval of 30 min between PIEBs; however, we are yet to determine the optimal interval for our program.

In 2020, Oluremi et al. published a control experiment comparing CEI and PIEB for labor analgesia [9]. In this trial, the lockout interval between the PCEA and PIEB was 10 min. In the CEI group, the lockout interval between the PCEAs was 10 min. They discovered that the ratio of PCEA attempts per hour was higher in the PIEB group than in the CEI group. Tien et al. also revealed comparable results in a retrospective investigation of 528 patients at an academic university medical facility [19]. Although the delay was 8 min in both the PIEB and CEI groups, the proportion of PCEA attempts was much greater in the PIEB group. In our department, the lockout interval was set to 15 min between each PIEB and PCEA and between PCEAs. Although the 15 min lockout interval is theoretically an ideal duration for long-acting local anesthetics to achieve a steady analgesic effect and is within the safety margin for parturients, it may increase the PCEA attempt/given ratio [20]. This indirectly leads to an increased anesthetic staff workload.

McKenzie et al. conducted a retrospective study after substituting background CEI with PIEB in their labor PCEA [21]. They found that substantially fewer females in the PIEB group needed a clinician rescue bolus during labor, but the time to first clinician bolus, and the number and amount of clinician boluses, did not differ between the two groups. Our findings contradict these previous findings for various reasons. First, in McKenzie’s study, both epidurals and CSEs were included in the analysis, whereas in our study, CSEs were eliminated. Although some anesthesiologists prefer dural puncture epidurals (DPEs), we did not use CSEs for painless labor due to the increased risk of pruritus, hypotension, simultaneous uterine tachysystole, and hypertonus [6,22]. To eliminate bias, 63 DPEs, and CSEs were excluded from the final analysis. Second, information on staff workload was gathered not just from electronic medical records, but also from the APS care diary. At 2 h intervals, the APS care diary recorded routine working content, such as medication refills, machine errors, and epidural tube blockade. Additional visits due to parturient complaints were also documented. We believe that this will provide a more detailed and accurate record of the anesthetic workload.

Furthermore, PIEB + PCEA settings and parameters are more complex than those of CEI + PCEA, CEI alone, or PCEA alone. Even if the PIEB + PCEA settings do not adequately meet the needs of users, they have benefits such as medication sparing and fewer side effects. However, as we discovered in this study, this may indirectly increase the workload of medical staff. The current findings suggest that we should consider including medical staff workload measurements as important indicators of self-quality assessment and improvement programs.

Afshan et al. conducted a prospective, randomized, double-blind study to determine the appropriate length of an epidural catheter in the epidural space [23]. The study revealed that a distance of 3–7 cm had a similar spread of local anesthetics within the epidural space, as confirmed by epidurography [23]. In our study, although the catheter length in the epidural space showed statistical differences (6.2 cm vs. 6.0 cm), both were within the suggested range. Moreover, the shorter distance in the PIEB group theoretically decreases the risk of unilateral blocks and the need for catheter adjustment.

This retrospective study has several limitations. In addition to the inherent limitation of not being able to establish a causal effect in a cross-sectional observational study like this one, there are also limitations from the clinical setting. First, after inserting the epidural catheter, our routine loading dose was only 6–10 mL of the local anesthetic. Compared with other studies, where the loading dose was typically 12–15 mL, the practices in other studies may expand the epidural space at the start and provide a better sensory block [9,18]. Second, the machine medication was levobupivacaine 0.125% with fentanyl 1.25 μg/mL, whereas the rescue medication was lidocaine 1%, making it difficult to compare the total amount of medication required between groups. Third, some detailed information, such as attempts/given ratios in PIEB or CEI devices, was excluded from the analysis because it is not typically recorded in standard medical records. Fourth, PIEB has some advantages during the second stage of labor, like shorter duration for primiparous women [24], but our data did not distinguish between the first and second stages of labor. Fifth, our study did not assess the degree of lower-limb weakness and rates of instrumental delivery. Finally, although removing CSE and DPE from the analysis increased homogeneity, it reduced generalizability.

## 5. Conclusions

In conclusion, we revealed that PIEB + PCEA used less analgesic medication to maintain labor analgesia; however, the complexity of its settings may increase the anesthetic staff workload when compared to CEI + PCEA.

## Figures and Tables

**Table 1:** Demographic characteristics of participants.

	CEI	PIEB	*t*	*p* ^a,b^
Sample size, *n*	214	272		
Age (yrs)	33.3 ± 4.6	33.0 ± 4.6	0.55	0.579
Weight (kg)	68.3 ± 9.5	68.6 ± 9.2	−0.48	0.630
BMI ^c^ (kg/m^2^)	26.4 ± 3.2	26.6 ± 3.2	−0.55	0.582
Gestational age (wks)	38.5 ± 1.2	38.5 ± 1.2	−0.32	0.752
Parity, n (%)				0.054
Primiparity	146 (68)	178 (65)		
Muliparity	68 (32)	94 (35)		
Cervical os (cm)	2.4 ± 1.5	2.5 ± 1.6	−0.19	0.847
Puncture level, *n* (%)				0.052
Lumbar 2–3	9 (4)	27 (10)		
Lumbar 3–4	198 (93)	234 (86)		
Lumbar 4–5	7 (3)	11 (4)		
Depth from the skin to epidural (cm)	4.8 ± 0.9	5.0 ± 1.1	−1.90	0.06
Depth of catheter insertion (cm)	6.2 ± 0.5	6.0 ± 0.7	2.84	0.01
Catheter fixation mark (cm)	11.0 ± 0.9	11.0 ± 1.2	0.11	0.92

Abbreviations: CEI, continuous epidural infusion; PIEB, programmed intermittent epidural bolus; BMI, body mass index. ^a^: *p*-value compares CEI vs. PIEB. ^b^: *t*-tests used to compare means and chi-square test used to compare proportions. ^c^: Body Mass Index.

**Table 2:** Secondary results between CEI and PIEB.

	CEI	PIEB	*t*	*p*
Total time to delivery (mins)	820 ± 531	814 ± 559	0.13	0.894
Total amount of medication (mL)	142 ± 92	122 ± 81	2.42	0.014
Average hourly dosage (mL/h)	11.0 ± 3.2	9.6 ± 2.2	5.71	<0.001
Shift from NSD to CS, *n* (%)	24 (10.1)	26 (8.7)		0.541
Satisfaction score (1–5)	3.9 ± 0.4	3.9 ± 0.4		0.327

**Table 3:** Two-way analysis of variance comparing the hourly medication usage in the time subgroups of CEI and PIEB groups.

Time	Groups				SS	df	MS	*F*	*p*
	CEI		PIEB						
	*n*	Mean (sd)	*n*	Mean (sd)					
<4	14	14.31 (3.57)	25	12.00 (1.98)	33.14	1	33.14	5.36	0.02
4–8	60	11.78 (2.76)	55	10.66 (2.26)	35.52	1	35.52	5.74	0.02
8–16	68	10.98 (3.71)	108	9.14 (2.04)	141.08	1	141.08	22.81	<0.001
16–24	40	10.10 (2.10)	49	8.87 (1.37)	47.83	1	47.83	7.73	<0.01
>24	32	9.60 (2.45)	35	8.53 (2.03)	19.01	1	19.01	3.07	0.08

## Data Availability

Due to the policy of the National Health Insurance Administration in Taiwan, the raw data of this study are not available.
